# Of Huge Mice and Tiny Elephants: Exploring the Relationship Between Inhibitory Processes and Preschool Math Skills

**DOI:** 10.3389/fpsyg.2015.01903

**Published:** 2016-01-07

**Authors:** Rebecca Merkley, Jodie Thompson, Gaia Scerif

**Affiliations:** Attention Brain and Cognitive Development Group, Department of Experimental Psychology, University of OxfordOxford, UK

**Keywords:** inhibitory control, interference, mathematics, preschool, magnitude comparison

## Abstract

The cognitive mechanisms underpinning the well-established relationship between inhibitory control and early maths skills remain unclear. We hypothesized that a specific aspect of inhibitory control drives its association with distinct math skills in very young children: the ability to ignore stimulus dimensions that are in conflict with task-relevant representations. We used an Animal Size Stroop task in which 3- to 6-year-olds were required to ignore the physical size of animal pictures to compare their real-life dimensions. In Experiment 1 (*N* = 58), performance on this task correlated with standardized early mathematics achievement. In Experiment 2 (*N* = 48), performance on the Animal Size Stroop task related to the accuracy of magnitude comparison, specifically for trials on which the physical size of dot arrays was incongruent with their numerosity. This highlights a process-oriented relationship between interference control and resolving conflict between discrete and continuous quantity, and in turn calls for further detailed empirical investigations of whether, how and why inhibitory processes matter to emerging numerical cognition.

## Introduction

Inhibitory processes allow the suppression of stimulus dimensions or motor responses according to task goals (e.g., Nigg, 2000; Friedman and Miyake, 2004). They belong to a set of overlapping and yet distinguishable processes, executive functions (EFs), that also comprise updating into working memory (“updating” henceforth) and shifting across task and stimulus dimensions (“shifting”; Miyake et al., 2000). Ample evidence suggests that all proposed executive constructs relate to numeracy: updating (St Clair-Thompson and Gathercole, 2006), inhibitory control (Clark et al., 2010), and shifting ability (Yeniad et al., 2013) have all been linked to mathematic achievement (Cragg and Gilmore, 2014; for reviews, see Bull and Lee, 2014). From a developmental point of view, relationships between these control processes and mathematics have been demonstrated for school aged-children (Bull and Scerif, 2001; St Clair-Thompson and Gathercole, 2006; Best et al., 2011; Van der Ven et al., 2012; Yeniad et al., 2013) and, in preschoolers, both concurrently and longitudinally (Bull et al., 2008, 2011; Clark et al., 2010, 2013; Steele et al., 2012). For example, in one of the largest and most comprehensive longitudinal studies of preschool executive control as a predictor of later math achievement to date, Clark et al. (2013) assessed EFs in 3-year-olds as a composite score based on measures of updating, shifting, and inhibitory control, following from earlier detailed investigations on the structure of executive control in preschoolers (Wiebe et al., 2008, 2011; Hughes et al., 2010). Clark et al. (2013) found that executive control at 3 years of age predicted math achievement 2 years later, over and above other measures including a standardized assessment of informal math skills (i.e., skills not explicitly taught in school).

Although these studies illustrate the strength and consistency of relationships between executive processes and math in early childhood, many authors acknowledge that questions about the precise nature of this relationship and potential causal mechanisms remained unanswered (e.g., Clark et al., 2013). Furthermore, both mathematics achievement and executive processes are multi-componential and the ways in which specific processes relate changes over developmental time (Cragg and Gilmore, 2014). For example, inhibitory control was found to be most strongly related to conceptual mathematics knowledge in adults, but most strongly associated with procedural knowledge in 11- to 14-year-old children (Gilmore et al., 2015). One way to disentangle these relationships is to use experimental manipulations to assess particular executive processes hypothesized to play a role in early maths achievement. Specifically, correlational investigations across multiple measures of EFs and mathematics need to be complemented with experiments, to elucidate precise relationships and highlight potential underlying causal mechanisms. These can in turn then be followed up through intervention regimes targeting causality most directly. Here, we focus on preschoolers' inhibitory control processes, because these have been explicitly postulated to act as limiting factors for emerging number abilities in early childhood (e.g., Houdé, 2000; Borst et al., 2012; Poirel et al., 2012; Gilmore et al., 2015). It is to these suggestions that we now turn.

### The role of inhibitory control in early numeracy

Failures of inhibition could account for many of Piaget's conclusions that the errors young children make are a result of immature concept knowledge or reasoning skills, both in general and in the context of numerical concepts (e.g., Houdé, 2000; Borst et al., 2012; Poirel et al., 2012). Particularly in relation to number development, failures on a conservation of number task may reflect a failure to inhibit the salience of information about length, to the detriment of number, rather than an inability to represent number itself. Cognitive development in the domain of number representation may therefore mean learning to ignore competing task-irrelevant dimensions of the stimulus. Indeed, a study in 3- to 5-year-old children from low-income families found that performance on a magnitude comparison task related to math achievement, but that this relationship was driven by trials that required inhibiting an irrelevant stimulus dimension (surface area) to select the larger numerosity (Fuhs and McNeil, 2013). This suggests that the ability to ignore irrelevant perceptual information and focus on number may explain why inhibitory control relates to early numeracy. Gilmore et al. (2013) found complementary results to Fuhs and McNeil (2013): in a sample of 4- to 11-year-old children, the relationship between magnitude comparison skills and math achievement only held for magnitude comparison trials that required the inhibition of dimensions in conflict with number. In a second experiment, 8- and 9-year-olds' math achievement was not uniquely predicted by magnitude comparison accuracy, over and above inhibitory control. The authors therefore argued that the relationship between the ability to compare magnitudes and math achievement could be accounted for by individual differences in inhibitory control (but see Keller and Libertus, 2015, for contradictory findings).

The debate on whether domain-specific skills like preschool magnitude comparison contribute to math achievement independently of inhibitory control remains open (e.g., Fuhs and McNeil, 2013; Gilmore et al., 2013; Keller and Libertus, 2015). Here, however, we instead tackle a complementary and not well-understood issue: why inhibitory skills *themselves* relate to mathematical skills in early childhood. There is indeed a likely precise cognitive reason why inhibitory control measures and magnitude comparison relate to each other concurrently. Non-symbolic magnitude tasks are typically used to measure the approximate number system (ANS), which is thought to be an innate cognitive system for representing approximate magnitude, with precision improving over development and with formal education (see Dehaene, 2011). In order to be sure that participants make judgments based on the number of stimuli in an array, and not on related features of continuous quantity, these visual properties (e.g., average diameter of the dots; the convex hull, the smallest contour around the dot area; and density, the average diameter divided by convex hull) are typically controlled for in non-symbolic arrays in numerical tasks (e.g., Halberda et al., 2008). This essentially leads to magnitude comparison tasks that heavily rely on inhibitory control, as on some trials number and continuous quantity are congruent whereas on others they are in conflict (e.g., Clayton and Gilmore, 2015). For example, Clayton and Gilmore (2015) systematically manipulated the inhibitory demands of a non-symbolic comparison task in order to investigate which factors influenced 7- to 9-year-old children's judgments. Specifically, they contrasted comparisons when discrete and continuous quantity variables were congruent with comparisons when discrete and continuous quantity dimensions were incongruent. Results revealed that both ANS acuity and inhibitory control skills influenced accuracy on the non-symbolic comparison task, with incongruent information associated with continuous quantity being more salient for larger numerosities than for smaller sets.

A similar non-symbolic numerical Stroop paradigm was used to test the interference of conflicting area information on number judgments and the reciprocal interference of number on area judgments earlier in development, in 4- to 6-year-old children (Rousselle and Noël, 2008). Participants also performed a Day/Night Stroop task, an established measure of inhibition in young children (Gerstadt et al., 1994). Results showed significant effects of congruity in both the area and numerosity judgment tasks. The size of the congruity effect of number on the area task increased with age, which supports the hypothesis that discrete numerosity becomes a more salient feature of the environment later in childhood (Mix et al., 2002; Cantrell and Smith, 2013; Leibovich and Henik, 2013). Number and other stimulus dimensions, such as area, usually covary in the environment. Therefore, discriminating continuous quantity typically leads to the same results as discriminating numerosity, so it could be that young children do not learn to distinguish continuous and discrete quantity until relatively late in childhood (Mix et al., 2002). Interference effects on the magnitude judgment tasks were not significantly correlated with interference effects on the Day/Night Stroop, which the authors took to suggest that non-numerical and numerical magnitude processing develop independently of inhibition. However, 4-and 6-year-olds' accuracy on the Day–Night task was quite high, suggesting potential ceiling effects and therefore reduced individual differences on that measure. Even when it was first introduced, Gerstadt et al. (1994) found that the Day–Night Stroop was quite difficult for children 3.5–4.5 years old but that it was comparably easy for 6 and 7-year-old. It could be that in the study by Rousselle and Noël (2008), this task was not a sensitive measure of inhibition in the majority of the sample and therefore was not useful for investigating the association between interference on magnitude comparisons and the development of inhibitory control.

### Inhibitory control and number sense across development

In summary, then, a working hypothesis is that inhibitory tasks requiring interference control, the process of suppressing a stimulus, or dimension of a stimulus, that requires a competing response to that required of the task instructions (Nigg, 2000), relate to early numeracy because early numerical tasks demand the ability to inhibit related non-numerical dimensions of stimuli. Indeed, there is an increasing body of evidence supporting the hypothesis that, even in adults, specific tasks that are traditionally construed as measurements of “number sense” (e.g., judgments of non-symbolic magnitudes) are influenced by stimulus dimensions such as area and density, regardless of how these parameters are controlled (Gebuis and Reynvoet, 2012; Szucs et al., 2013). Gebuis and Reynvoet (2012) manipulated the average diameter of the dots, the convex hull, and density of dot arrays so that each feature was not correlated with increasing numerosity and had adults perform a numerical estimation task on these stimuli. The size of visual cues influenced estimates despite the fact that the cues separately did not correlate with numerosity, supporting the authors' hypothesis that information about numerosity is obtained by combining multiple visual cues. Furthermore, adults are susceptible to interference from area on a numerical Stroop task (Hurewitz et al., 2006). Therefore, numerical competence may reduce, but not eliminate entirely, the requirement to inhibit perceptual dimensions of stimuli when they conflict with numerosity. In a related vein, a main effect of congruency of number on an area judgment task was found in adults' performance on a number/size Stroop paradigm (Nys and Content, 2012). The authors argued that adults do automatically extract numerosity from non-symbolic arrays and that this interferes with area judgments. An arithmetic measure was also included in the study and did not correlate with overall accuracy on the numerical comparison task but did correlate with the effect size of the interference of number on the area comparison task, suggesting that, in adults, math achievement is associated with the ability to extract information relevant to number in non-symbolic arrays.

The role of inhibitory control in mathematics achievement is of particular interest given the mixed evidence for the relationship between non-symbolic magnitude comparison and math achievement from primary school into adulthood (see De Smedt et al., 2013, for a review). While symbolic magnitude processing has consistently been shown to correlate with formal mathematics performance, conflicting findings have been reported concerning non-symbolic magnitude processing. One explanation for the varied results is that non-symbolic comparison tasks vary in terms of control of visual parameters, length of stimulus display, and even which outcome measure is used. ANS precision can be operationalized as overall accuracy on a comparison task, the Weber fraction estimate of performance, or ratio effects on accuracy or reaction time and these different indices of performance do not always correlate strongly with each other (Price et al., 2012; Gilmore et al., 2014). As non-symbolic tasks measure inhibitory control in addition to number sense (Clayton and Gilmore, 2015), it could be that the observed relationships between non-symbolic comparison and maths achievement are driven by inhibitory control (Fuhs and McNeil, 2013; Gilmore et al., 2013; but cf. Keller and Libertus, 2015). A recent meta-analysis of the relationship between non-symbolic comparison and mathematics achievement found that this relationship was overall weak, but strongest for children younger than six (Fazio et al., 2014). Similarly, younger children are more susceptible to congruity effects on non-symbolic comparison tasks than older children and adults are (Szucs et al., 2013). This suggests that the inhibitory demands of this task are strongest for young children, and this in turn may explain why non-symbolic comparison and maths relate most strongly in this age group.

## Experiment 1

The current study was designed to test the hypothesis that inhibitory control, specifically interference control, is relevant to numeracy skills prior to the start of formal education. An Animal Size Stroop task (Szucs et al., 2009; Bryce et al., 2011) was modified to be appropriate for preschoolers. Participants had to decide which of two animals was larger in real life and the size of the images was manipulated so that, on incongruent trials, the size of the animal image was in conflict with its size in the real world. A different version of an Animal Stroop was previously developed by Wright et al. (2003) in which children were required to name the animal stimuli, including incongruent trials where stimuli had heads and bodies of different animals. However, in the current experiment size congruency was of interest as this stimulus dimension may be especially relevant to early maths skills. A task similar to the current one is also part of the Cognitive Assessment System (Naglieri and Das, 1997), although participants are required to provide verbal responses.

The goal of the current study was to use this measure to elucidate specific relationships between inhibitory control and early numeracy skills in young children. Previous studies (specifically, Fuhs and McNeil, 2013; Gilmore et al., 2013; Keller and Libertus, 2015) have investigated whether inhibitory control accounts for the observed relationship between non-symbolic comparison and mathematics achievement (i.e., tested whether relationships between non-symbolic comparisons and maths achievement hold over and above inhibitory skills as a potential confound). Here, we focused instead on why inhibitory control may be important for early numeracy. Based on the hypothesis that ignoring irrelevant stimulus dimensions plays a role in mathematics achievement, we predicted performance on the Animal Size Stroop to be associated with math achievement, as measured by a standardized measure of mathematics in young children. We also expected Animal Size Stroop performance on high conflict trials to be associated, more precisely, with non-symbolic magnitude comparison, given the inhibitory demands of attending to discrete quantity in the face of competing continuous properties.

### Method

#### Participants

Seventy children recruited from local nurseries and schools participated. Following ethical approval from the Central University Research Ethics Committees (University of Oxford), informed consent was obtained from parents. Twelve children were excluded for missing data for two or more measures. Eight of the twelve excluded children were 3-year-olds, which suggests that the tasks may be more difficult for the youngest children, but the excluded children did not differ from the rest of the sample in terms of gender, verbal IQ or visual-spatial IQ. The final sample used in the analyses included 58 children ranging in age from 36 to 72 months (*M* = 51.97, SD = 10.98), of which 29 were female. According to neighborhood summaries of indices of deprivation, the schools we recruited from were in neighborhoods ranked relatively low in deprivation (Office for National Statistics, 2010). Neighborhoods from which we recruited ranked in the 97th percentile of least deprived, 80th percentile, 48th percentile, and 31st percentile, respectively.

#### Materials

All computer games were presented with E-Prime 1.0 software on an Elo AccuTouch 17-inch touchscreen monitor.

##### Animal size stroop task

The stimuli were taken from a set of colored Snodgrass and Vanderwart pictures (Rossion and Pourtois, 2004). They were chosen based on results from a pilot task in which 3- to 4-year-old children played a zoo sorting game and sorted 17 animal pictures (7.5 by 4.5 cm) into a small or large cage based on their real life size. Four large animals (elephant, horse, cow, and lion) and four small animals (frog, ladybird, mouse, and rabbit) that were sorted correctly by the majority of children were selected for inclusion in the Stroop task. Participating children also completed the sorting task as part of the experimental protocol in order to assess their knowledge of animals' sizes. Children who failed to correctly sort at least six of the eight animals were excluded from the study, as the size congruency manipulation on the Animal Size Stroop task was dependent on animal size knowledge.

**Figure 1:**
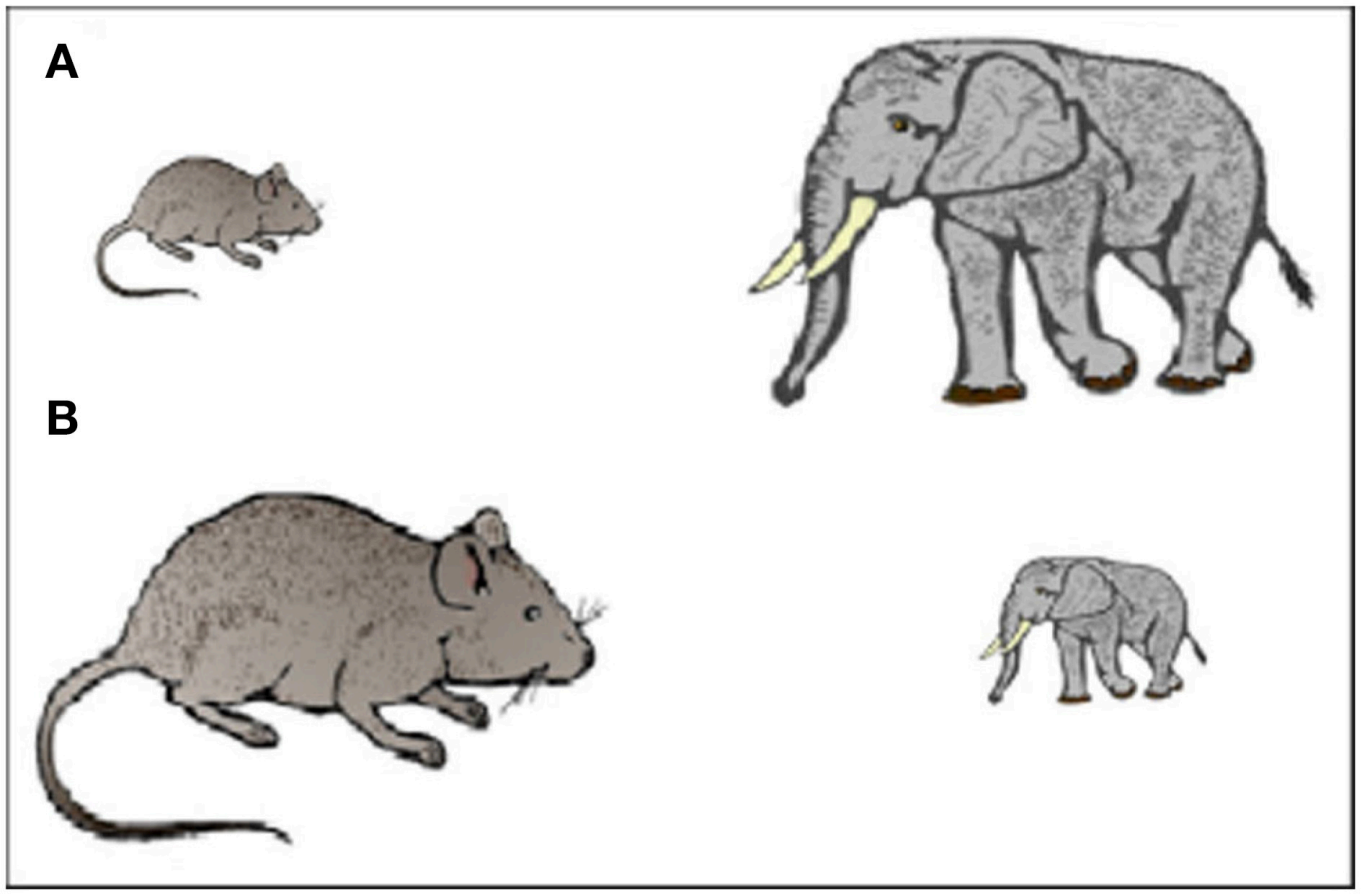
**Example stimuli. (A)** Congruent condition. **(B)** Incongruent condition.

Children were instructed to choose which of two animals was the largest in real life, whilst ignoring the differing sizes of the animal images on the computer screen. The two animals were displayed on the screen with a smaller image (6 by 4 cm) on one side and a larger image (15 by 10 cm) on the other (see Figure 1). The response area was identical for each picture (17 × 14 cm) and stimuli were displayed for up to 5000 ms. Correct responses were reinforced with a smiley face displayed for 100 ms, whereas incorrect responses were followed by a blank display. Congruency, response side, and animal size were counterbalanced and trials were displayed in random order.

##### Test of early math achievement—3

Early numeracy skills, including counting, cardinality, symbolic number knowledge, magnitude comparison, and arithmetic, were assessed with the Test of Early Math Achievement—3 (TEMA-3; Ginsburg and Baroody, 2003). The TEMA-3 has 72 items and testing was discontinued when children reach a ceiling of five consecutive failed items. Entry point varies by age, but a basal of five consecutive correct items must be reached. The raw score was the number of items correct below ceiling and all items below the basal were counted as correct.

##### Magnitude comparison task

**Figure 2:**
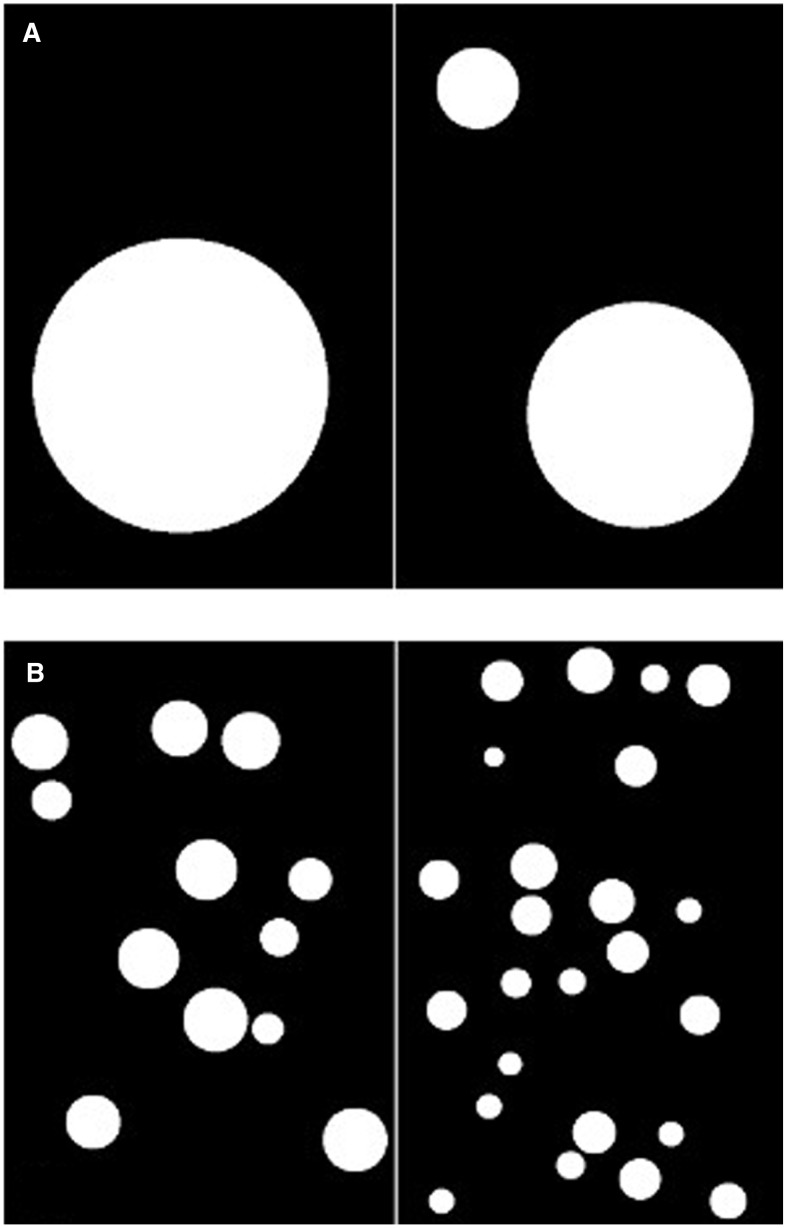
**Example stimuli. (A)** Small number condition. **(B)** Large number condition.

A computerized non-symbolic magnitude comparison task was used to measure ANS acuity. Children had to pick the larger of two dot arrays and were instructed to help Bob the Builder judge the hole digging competition. In order to test whether set size influenced magnitude comparison, one block of trials required small comparisons (1–3), and the other large comparisons (10–39; see Figure 2). The ratio between each comparison was small (0.33), medium (0.5), or large (0.67). Order of block presentation was counterbalanced and there were 24 trials per block. The dots were presented for a maximum of 1200 ms to discourage counting. A blank screen followed dot presentation for an additional 1800 ms, which allowed for a total response window of 3000 ms. Contour, area, density, and brightness of the stimuli were controlled for and the area of individual dots was randomized across items and numerosity. Specifically, dot arrays were created using the method of Price et al. (2012): total surface area was equated across arrays on half of the trials and total perimeter was equated in the other half of the trials. Therefore, participants could not consistently base judgments on one type of visual cue associated with continuous quantity.

##### British ability scales—II

The Pattern Construction Subscale of the British Ability Scales-II (PC-Subscale, BAS-II; Elliot et al., 1996) was used to assess non-verbal IQ. Children used colored foam squares and patterned cubes to copy patterns presented in a book. Scores were based on response time and accuracy.

##### British picture vocabulary scale—III

Verbal IQ was measured with the British Picture Vocabulary Scale (BPVS-III; Dunn and Dunn, 2009), a measure of receptive vocabulary. Children were shown four pictures and instructed to point to the picture that corresponded to the word said by the experimenter. The raw score was the total number of items correct and the task was administered until a child made eight or more errors on one set of 12 items.

### Procedure

Each child was tested in a quiet area for approximately 1.5 h over the smallest number of possible sessions, over multiple days if needed to maximize completion rates, and given regular breaks. The data included in this experiment are a subset from a larger battery of tasks, administered in a counterbalanced order across children. All sessions took place with an experimenter at the school or nursery and children were allowed to choose a sticker at the end of each session.

#### Analysis

Raw scores of standardized measures were used in the analyses as not all included tasks were standardized. Overall accuracy was used as the dependent measure for magnitude comparison and Stroop tasks in the analyses as well as accuracy separated by congruence condition on the Stroop as this method was used in previous studies (Fuhs and McNeil, 2013; Gilmore et al., 2013). Furthermore, Gilmore et al. (2014) showed that overall accuracy was the most reliable measure of ANS acuity on magnitude comparison tasks in children. Analyses on reaction time data are not reported because low accuracy led to a limited number of valid trials contributing to these analyses.

### Results

#### Descriptive statistics

Statistical analyses were performed with SPSS (release version 20.0.0). Descriptive statistics separated into age categories are reported in Table 1. One child had only just turned 6 years old in the month they were tested and was therefore included with the other 5-year-olds. Age in months was significantly correlated with all measures and we therefore included it as a categorical variable in all analyses. There were no significant gender differences on any of the measures.

**Table 1:** **Descriptive statistics separated by age**.

		**3 years old**		**4 years old**		**5 years old**	
**Task**	**Measure**	**Mean (SD)**	***N***	**Mean (SD)**	***N***	**Mean (SD)**	***N***
TEMA	Raw score	9.69 (5.61)	26	15.07 (4.83)	14	28.11 (7.32)	18
BAS	Raw score	7.28 (5.95)	25	12.35 (8.15)	14	25 (6.78)	18
BPVS	Raw score	39.92 (8.59)	25	51.85 (11.46)	13	66.44 (8.19)	18
Animal	Accuracy (congruent)	86.06% (13.91%)	22	89.64% (13.33%)	12	94.59% (8.79%)	17
Size	Accuracy (incongruent)	72.27% (32.39%)	22	66.72% (27.38%)	12	89.76% (18.77%)	17
Stroop	Median RT (congruent)	1471.3 (405.69)	22	1243.04 (236.61)	12	1097.41 (219.56)	17
	Median RT (incongruent)	1709.84 (301.99)	20	1472.67 (309.44)	12	1231.35 (221.2)	17
Magnitude	Accuracy (small)	48.24% (22.86%)	22	48.18 (18.52%)	13	78.53% (21.16%)	17
Comparison	Accuracy (large)	51.46% (14.26%)	23	43.31% (16.12%)	12	68.41% (22.62%)	17
	Median RT (small)	1502.73 (514.62)	22	1156.62 (483.59)	13	1245.03 (413.06)	17
	Median RT (large)	1274.23 (482.05)	23	1045.21 (316.65)	12	1087.15 (385.78)	17

*TEMA, Test of Early Mathematics Achievement—III; BAS, British Abilities Scale; BPVS, British Picture Vocabulary Scale*.

#### Animal size stroop

Two children failed to complete the task and five children sorted less than 75% of the animals correctly in the sorting task and were excluded from the analysis. Therefore, data from 51 participants were included in the analysis. Kolmogorov–Smirnov tests revealed that neither incongruent nor congruent accuracy scores were normally distributed. In order to deal with these violations, but still test the hypotheses set out in the introduction, statistically significant effects were also tested using the appropriate equivalent non-parametric statistics. Unless reported otherwise, parametric and non-parametric statistics were consistent with each other. A repeated measures analysis of variance (ANOVA) was run on the accuracy data with Congruency as a within-subjects factor and Age in years as a between subjects factor. Results revealed a significant main effect of congruency, *F*(1, 48) = 15.19, *p* < 0.001, $\eta_{p}^{2}$ = 0.24, driven by higher accuracy on congruent (*M* = 90.1%, SE = 1.8%) than incongruent trials *(M* = 76.3%, SE = 3.9%). There was also a main effect of Age, *F*(1, 49) = 3.4, *p* = 0.042, $\eta_{p}^{2}$ = 0.12. Bonferroni-corrected *post hoc* tests showed that 5-year-olds' accuracy *(M* = 92.2%, SE = 4.2%) showed a trend toward being significantly higher than 3-year-olds' accuracy (*M* = 79.2%, SE = 3.7%), *p* = 0.07, and there was no significant differences between 5 and 4-year-olds' accuracy *(M* = 78.2%, SE = 5%), *p* = 0.11.

#### Magnitude comparison

Performance on this task was quite low (see Table 1), in part because of missed responses due to the speeded aspect of the task. Overall mean accuracy on large comparisons was 55.12% (SD = 20.05) and a one-sample *t*-test showed that this was not significantly different from chance performance, 50%, *t*(51) = 1.842, *p* = 0.071. Only 12 children had mean accuracies greater than 50% for both large and small comparisons and therefore using an accuracy cut off to limit analyses to children performing better would exclude the majority of the data set.

Kolmogorov–Smirnov tests showed that accuracy was not normally distributed in any condition except in the small comparison, large ratio condition. With caution, a repeated measures ANOVA was run with comparison Size and Ratio as within-subjects factors and Age in years as a between-subjects factor. Results revealed a significant main effect of Age, *F*(2, 46) = 14.46, *p* < 0.001, $\eta_{p}^{2}$ = 0.386. Bonferroni-corrected pairwise comparisons showed that 5-year-olds' accuracy (*M* = 73.5%, SE = 3.8%) was significantly higher than both 4-year-olds' (*M* = 45.8%, SE = 4.5%) and 3-year-olds' (*M* = 50%, SE = 3.5%) accuracy (*p*s < 0.001), and there was no significant difference between 3 and 4-year-olds, *p* = 1. There was a significant interaction between Size, Ratio, and Age *F*(4, 92) = 2.87, *p* = 0.027, $\eta_{p}^{2}=0.111$. An analysis of simple main effects revealed this was driven by a significant main effect of Size on medium ratio trials for 5-year-olds, *F*(1, 46) = 7.51, *p* = 0.009, $\eta_{p}^{2}$ = 0.14. This effect is difficult to interpret as it was only seen on medium ratio trials, yet there were no significant main effects of ratio. Given the overall low performance on this measure, it was excluded from subsequent analyses.

#### Predicting early mathematics achievement

Bivariate and partial correlations between measures are reported in Table 2. Non-parametric bivariate correlations were also conducted, given the previously reported violations of parametric assumptions, and unless explicitly stated, were consistent with the parametric counterpart. Parametric statistics were also interpreted with caution throughout. When age was partialled out, TEMA score was significantly correlated with BAS score, *r*(54) = 0.463, *p* < 0.001, and also with accuracy on the congruent and incongruent conditions of the Animal Size Stroop, congruent condition *r*(47) = 0.379, *p* = 0.007, incongruent condition, *r*(47) = 0.358, *p* = 0.011.

**Table 2:** **Spearman's bivariate correlations (above diagonal) and Pearson's partial correlations controlling for age in months (below diagonal)**.

**Measure**	**1**	**2**	**3**	**4**	**5**	**6**
1. Age	–	0.739^**^	0.792^**^	0.832^**^	0.351^*^	0.38^*^
2. BAS		–	0.702^**^	0.768^**^	0.256	0.43^**^
3. BPVS		0.254^*^	–	0.718^**^	0.321^*^	0.289^*^
4. TEMA		0.463^**^	0.215	–	0.504^**^	0.52^**^
5. AS congruent		0.048	0.131	0.379^**^	–	0.535^**^
6. AS incongruent		0.178	0.033	0.358^*^	0.404^**^	–

* p < 0.05;

** *p < 0.01. BAS, British Abilities Scale; BPVS, British Picture Vocabulary Scale; TEMA, Test of Early Mathematics Achievement—III; AS, Animal Size Stroop*.

Hierarchical linear regression models were run in order to test whether inhibitory control, as measured by performance on the Animal Size Stroop, predicted TEMA score. Age alone accounted for approximately 71% of the variance in TEMA raw scores (adjusted *R*^2^ = 0.705), *t* = 11.72, *p* < 0.001 (see Table 3). Overall accuracy on the Animal Size Stroop was a significant predictor of TEMA score above and beyond the variance that could be accounted for by age and BAS raw score, *t* = 2.78, *p* = 0.008, and the model had predictive validity, *F*(3, 46) = 72.98, *p* < 0.001, adjusted *R*^2^ = 0.815.

**Table 3:** **Regression models predicting TEMA scores**.

	**Variable**	**β**	**Adjusted *R*^2^**
Model 1	1. Age (months)	0.843^**^	0.705
Model 2	1. Age	0.53^**^	0.767
	BAS	0.4^**^	
Model 3	1. Age	0.517^**^	0.815
	2. BAS	0.348^**^	
	3. Stroop accuracy	0.188^**^	

** *p < 0.01. BAS, British Abilities Scale*.

### Discussion

The significant effects of congruency on Animal Size Stroop accuracy suggested that this task was an effective measure of inhibitory control in preschoolers. Although there were main effects of age, with older children performing better than younger children, the older children also experienced conflict effects, suggesting that the task effectively measures inhibition in this specific age-range (3- to 5-year-olds). A limitation of the Animal Size Stroop is that it requires knowledge of real world animal sizes and preschoolers' knowledge varies depending on their experience, even for the animals that were selected to be highly familiar to the youngest children in our target age range. Accordingly, some 3-year-olds were excluded from the current study because they lacked sufficient animal size knowledge. Individual differences in young children's animal size knowledge may be associated with variability in caregiver instruction and a potential alternative that cannot be ruled out by the current study is that children who have greater animal size knowledge have also received more instruction in other domains, including number. As children who did not meet the minimum knowledge requirements were excluded, and significant congruency effects were observed, participating children did have enough animal size knowledge to experience interference from incongruent size information on the task.

Mean accuracy on the non-symbolic magnitude comparison task was below chance level for both 3- and 4-year-olds, and 5-year-olds performed significantly better than the younger children. While some published studies do not report such high failure rates in this age group (e.g., Mazzocco et al., 2011; Keller and Libertus, 2015), a recent study found that in a large sample of children in kindergarten, only 57% showed statistically reliable ratio effects on a non-symbolic comparison task, even after excluding children who failed to perform above chance level (Lyons et al., 2015). Previous studies that have excluded children whose performance did not show significant effects of ratio may therefore have been failing to capture performance that is representative of the wider population, and especially of the youngest children. The current findings are also convergent with those of previous research showing that young children had difficulty choosing the larger of two non-symbolic arrays when perceptual features such as surface area were in conflict with numerosity (Rousselle et al., 2004; Negen and Sarnecka, 2014). This lends further support to the hypothesis that young children only learn to distinguish discrete and continuous quantity through experience with number (Mix et al., 2002; Rousselle and Noël, 2008; Leibovich and Henik, 2013; Negen and Sarnecka, 2014). Five-year-olds in the United Kingdom attend compulsory education, and therefore had more formal instruction about number. This may also explain why 5-year-olds' accuracy on the magnitude comparison task was significantly higher than 3- to 4-year-olds'. It could be that having more experience with number facilitates interference of irrelevant cues associated with continuous quantity.

As predicted, performance on the Animal Size Stroop task was correlated with performance on a standardized measure of mathematics achievement. This relationship remained significant when age and non-verbal IQ were accounted for, and suggested that the ability to inhibit stimulus dimensions specifically associated with number is related to early numeracy skills beyond simply judging the larger of two non-symbolic arrays. The TEMA includes measures of counting and cardinality and does include a non-symbolic comparison item, but the stimuli are not controlled for area and so do not create conflict. Fuhs and McNeil (2013) found a similar relationship between their inhibitory control measure and TEMA score in preschoolers from a low SES background, when accounting for verbal IQ as measured by receptive vocabulary. Furthermore, they found that when they entered inhibitory control into their regression models, ANS acuity was no longer a significant predictor of TEMA score, and that without the inhibitory control measure, ANS acuity was a significant predictor, but that this relationship was driven by incongruent comparison trials. Those findings remain hotly debated, with a recent study in preschoolers of mid-to-high SES showing robust correlations between non-symbolic magnitude comparison skills and TEMA scores, even when controlling for individual differences in inhibitory control (Keller and Libertus, 2015).

In the current study, we did not aim to resolve the controversy on whether magnitude comparison accuracy is an independent predictor of early mathematics achievement, but rather targeted the role played by inhibitory control skills themselves. Accuracy on the Animal Size Stroop was a significant predictor of TEMA score, even after controlling for age and non-verbal IQ. Poor performance on the non-symbolic comparison task prevented us from assessing its relationships with our inhibitory control task, and therefore we could not test the hypothesis that inhibitory control is required for inhibiting interfering cues associated with continuous quantity. We therefore manipulated the inhibitory demands of a non-symbolic comparison task directly, to explore this hypothesis in Experiment 2.

## Experiment 2

Performance on the magnitude comparison task in Experiment 1 was very low and main effects of ratio did not reach significance. The magnitude comparison task in Experiment 2 was therefore designed to be easier for young children than the previous one was, by manipulating stimulus parameters and allowing for slower responses. A previous study in preschoolers used stimuli in which the overall surface area was controlled for, but the size of individual objects in an array was homogeneous, and found that magnitude comparison performance was significantly above chance level (Wagner and Johnson, 2011). In this experiment, we also kept the size of individual dots constant within arrays in order to make numerosity more salient than other visual features. Additionally, we aimed to test more precisely whether the relationship between inhibitory control and magnitude comparison is driven by congruence of stimulus dimensions and included a manipulation of surface area similar to that of previous number/area Stroop tasks used with young children (Rousselle et al., 2004; Rousselle and Noël, 2008; Fuhs and McNeil, 2013; Keller and Libertus, 2015). We hypothesized that greater inhibitory control would be required under conditions of conflict between number and other stimulus dimensions. We predicted that Animal Size Stroop performance would most strongly predict magnitude comparison abilities under conditions of conflict between number and dot size (when area across distinct numerosities was equated, putting other dimensions, e.g., average dot size, in conflict with number, “incongruent trials” henceforth), compared to the situation in which number and area were allowed to covary (“congruent trials” henceforth), providing redundant information.

### Method

#### Participants

Sixty-seven children between the ages of three and six recruited from local nurseries and schools participated. As above, the Central University Research Ethics Committees approved the study and informed consent was obtained from parents. Five children were excluded due to insufficient animal knowledge, four children were excluded for failing to complete tasks, nine were excluded for not following task instructions, and one was excluded because of a diagnosis of a learning disorder. All of the excluded children were 5 years old or younger. The final sample used in the analyses included 48 children ranging in age from 39 to 80 months (*M* = 63, SD = 13), of which 31 were female.

#### Materials

All computer games were presented with E-Prime 2.0 software. Children in nurseries played on an Elo AccuTouch 17-inch touchscreen monitor and children in schools played on a 13.3-inch Toshiba laptop and used buttons on the keyboard to make responses.

##### Magnitude comparison

**Figure 3:**
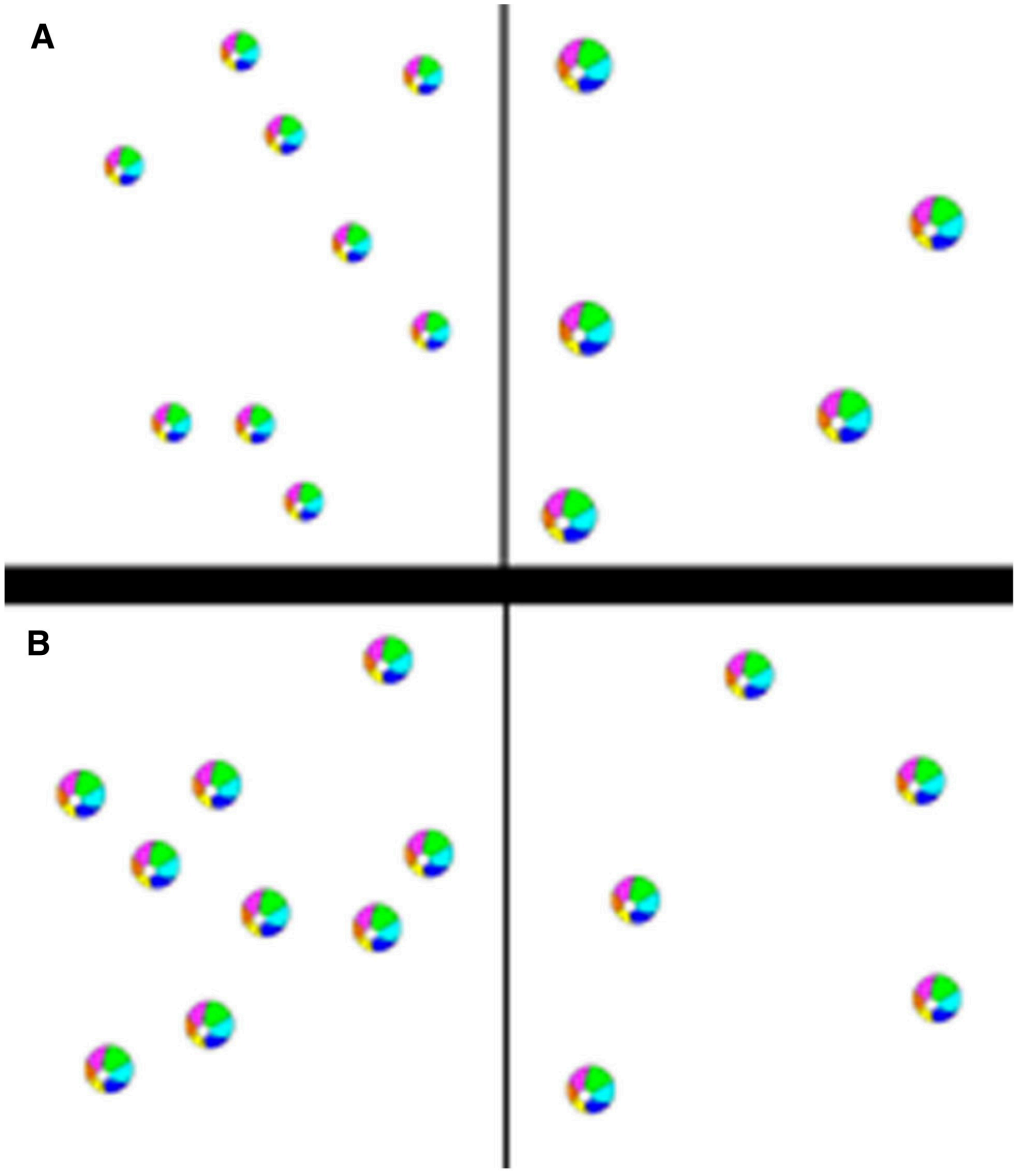
**Example stimuli. (A)** Incongruent condition. **(B)** Congruent condition.

Children were asked to choose who had more, a hippo character or a moose character and to indicate the side with more by either touching the monitor or pushing a button. There was a 500 ms fixation screen followed by a 2500 ms stimulus display screen and then an unlimited response window. Stimulus display was timed in order to prevent counting but children were able to respond afterward in order to avoid missed trials due to slow processing speed. Quantities ranged from 1 to 9 and pairs were chosen from ratios 0.25, 0.5, and 0.75, and we would expect magnitude comparisons to be more accurate and faster for pairs of quantities that yielded a small ratio (e.g., 1 and 4). Pairs were randomly presented and the side of the screen on which the largest quantity appeared was counterbalanced. In one block, all of the items were the same size, so that the array with the larger number of items also had a greater total surface area (see Figure 3A, congruent). In the other, the total surface area of the stimuli was equated across arrays so that the items were smaller in size in the array with the larger number of items (see Figure 3B, incongruent). We predicted that a greater degree of inhibitory control would be needed to compare numerosity in the incongruent compared to the congruent trials. Presentation of these two conditions was blocked here to facilitate explaining, especially to the youngest children, that their task throughout was to report which side of the display contained “more objects'” regardless of their visual characteristics. Block order was counterbalanced between participants. Both dots and real circular objects were used to retain task engagement in younger children, but as the particular stimuli did not affect accuracy or speed, analyses were carried out collapsing across stimulus types.

#### Procedure

The data included in this experiment are a subset from a larger battery of tasks. Each child was seen for two sessions lasting approximately 1 h in total. All sessions took place with an experimenter at the school or nursery and children were allowed to choose a sticker at the end of each session. The Animal Stroop task was administered in the same way as in Experiment 1 but the animal knowledge test was only administered to children who were in nursery.

#### Analysis

As in Experiment 1, accuracy was used as the dependent measure for both Animal Size Stroop, and non-symbolic magnitude comparison. Accuracy has consistently been used as the dependent measure for non-symbolic comparison (e.g., Clayton and Gilmore, 2015; Gilmore et al., 2015), whereas reaction time has been used as the dependent measure of an Animal Size Stroop task for older children (Gilmore et al., 2015). In the current study, reaction times would be more difficult to interpret given the untimed nature of the task and the large discrepancy in performance between younger and older children. Like in Experiment 1, age was treated as a categorical variable, but here age was split into two groups: three and 4-year-old children were categorized as “younger children,” whereas 5 and 6-year-old children were considered “older children,” as this dealt better with violations of parametric statistics assumptions.

### Results

#### Animal size stroop

**Figure 4:**
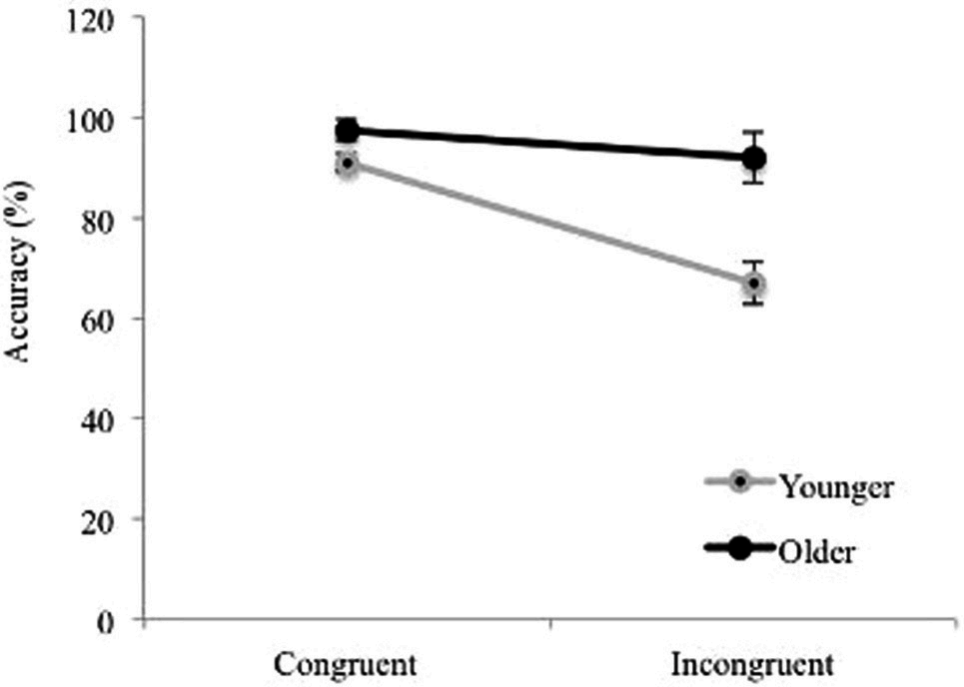
**Animal Stroop accuracy separated by Age and Congruency**. Error bars represent standard error.

Accuracy data on the Animal Size Stroop were analyzed with an ANOVA with Congruency as a within subjects factor and Age as a between subjects factor. Overall accuracy was used as the dependent measure as there was no significant difference between comparisons within and outside of the subitizing range, *t*(58) = 0.49, *p* = 0.626. Results revealed significant main effects of Age, *F*(1, 46) = 16.78, *p* < 0.001, $\eta_{p}^{2}$ = 0.27 and Congruency, *F*(1, 46) = 20.26, *p* < 0.001, $\eta_{p}^{2}$, = 0.31. There was also a significant interaction between Age and Congruency *F*(1, 46) = 8.15, *p* = 0.006, $\eta_{p}^{2}=0.15$ (see Figure 4). An analysis of simple main effects revealed that, for congruent trials, older children (*M* = 97.4%, SE = 1.8%) were significantly more accurate than younger children (*M* = 91%, SE = 2.1%), *F*(1, 46) = 5.49, *p* = 0.024, $\eta_{p}^{2}$ = 0.11, and this was also true for incongruent trials *(M*_*older*_ = 92%, SE = 4.2%; *M*_*younger*_ = 67%, SE = 5%), *F*(1, 46) = 14.5, *p* < 0.001, $\eta_{p}^{2}$ = 0.24. Furthermore, accuracy was significantly higher for congruent than incongruent trials in younger children, *F*(1, 46) = 23.19, *p* < 0.001, $\eta_{p}^{2}$ = 0.34, *p* < 0.001, but the difference did not reach significance in older children. Wilcoxon Signed Ranks tests confirmed non-parametrically a significant difference between accuracy on congruent and incongruent conditions for younger children, *Z* = −3.06, *p* = 0.002, and also revealed a significant difference between accuracy on congruent and incongruent conditions for older children as well, *Z* = −2.09, *p* = 0.037, although this effect was weaker than that in the younger children.

#### Magnitude comparison

**Figure 5:**
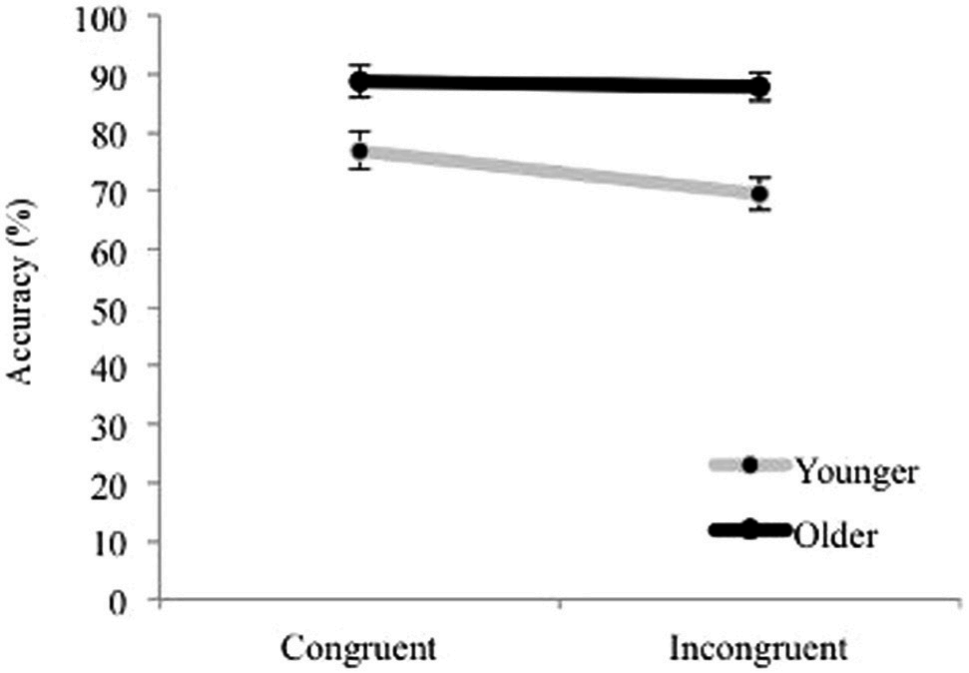
**Magnitude comparison accuracy plotted by Age and Congruency**. Error bars represent standard error.

The effect of block order did not reach significance, *F* < 1. Accuracy data on the magnitude comparison task was analyzed with an ANOVA with Congruency and Ratio (small, medium, or large) as within subjects factors and Age as a between subjects factor. The Greenhouse–Geisser correction was used for the effects of Ratio as the assumption of sphericity was violated. Results revealed expected significant main effects of Congruency, *F*(1, 46) = 4.7, *p* = 0.035, $\eta_{p}^{2}$ = 0.09, driven by higher accuracy for congruent (*M* = 82.8%, SE = 1.9%) than incongruent trials (*M* = 78.6%, SE = 2.1%), and Ratio, *F*(2, 92) = 51.81, *p* < 0.001, $\eta_{p}^{2}$ = 0.53. Accuracy was higher for small ratio trials (*M* = 89.3%, SE = 1.5%) than medium ratio trials (*M* = 82.7%, SE = 2.2%), and higher for medium compared to large ratio trials *(M* = 70%, SE = 2.4%), *p*s < 0.001. There was also a main effect of Age, *F*(1, 46) = 19.4, *p* < 0.001, $\eta_{p}^{2}=$ 0.3, driven by better performance by older children *(M* = 88.2%, SE = 2.2%) than by younger children (*M* = 73.1%, SE = 2.6%; see Figure 5). None of the interaction effects reached significance. Kolmogorov–Smirnov tests revealed that neither incongruent nor congruent accuracy scores were normally distributed and Box's M was significant, suggesting caution when interpreting parametric statistics. A Mann–Whitney test confirmed non-parametrically that there was a significant age difference, *Z* = −3.88, *p* < 0.001. Of note, a Wilcoxon Signed Ranks Test showed that the difference between accuracy on congruent and incongruent trials was less robust when tested non-parametrically, as it failed to reach significance, *Z* = −1.43, *p* = 0.153. However, a Pearson's bivariate correlation revealed a moderate negative correlation between age in months and the difference between accuracy on congruent trials compared to incongruent trials (i.e., conflict score) *r*(46) = −0.31, *p* = 0.034, suggesting that younger children were more sensitive to the congruency between number and area than older children were.

#### Relationships between inhibitory control and number sense

Animal Size Stroop conflict scores, i.e., the difference in accuracy between conditions on the Animal Size Stroop, were significantly and negatively correlated with accuracy on incongruent trials in Magnitude Comparison, *r*(45) = −0.351, *p* = 0.015, even when accuracy on the congruent condition of the magnitude comparison was partialled out. In other words, larger susceptibility to interference on the Animal Size Stroop was associated with lower accuracy on the incongruent magnitude comparison trials. However, the reverse did not hold: Accuracy conflict scores on the Animal Size Stroop task were not significantly correlated with accuracy on congruent magnitude comparison trials when accuracy on incongruent magnitude comparison trials was partialled out, *r*(45) = 0.062, *p* = 0.678, suggesting a specific relationship between conflict scores on the Animal Size Stroop and the higher conflict condition in the magnitude comparison task.

### Discussion

Ratio effects reached significance on this modified magnitude comparison task, contrary to what was seen with the previous version of the task in Experiment 1. This suggests that children were basing their judgments on the number of stimuli and not solely on interfering perceptual information. Similarly to what was found in previous studies (Fuhs and McNeil, 2013; Gilmore et al., 2013), performance on magnitude comparison trials when size was in conflict with number was correlated with a measure of inhibitory control, but performance on trials when size and number were correlated was not. Specifically, performance on the incongruent trials was associated with the ability to inhibit irrelevant information about stimuli size. These results support the argument that non-symbolic comparison tasks are inhibitory control tasks when visual features are manipulated (Gilmore et al., 2013; Clayton and Gilmore, 2015). There was no significant difference in overall accuracy between the incongruent and congruent trials on the magnitude comparison task according to more conservative non-parametric tests, similar to what was found in Keller and Libertus (2015) Experiment 1. In other previous studies (Rousselle and Noël, 2008; Fuhs and McNeil, 2013), significant differences in accuracy were found between conditions, but additional manipulations were built into those experiments that pitted continuous quantity and number even further in conflict. In the current study, the difference between conditions did negatively correlate with age, suggesting that children become less susceptible to conflicting size information with age and formal education. This finding supports the hypothesis that children become better at discerning discrete quantity with age and experience (Mix et al., 2002; Rousselle and Noël, 2008; Leibovich and Henik, 2013; Negen and Sarnecka, 2014). Indeed, the difference between congruent and incongruent conditions on the Animal Size Stroop was significantly correlated with incongruent trials on the magnitude comparison task, suggesting that inhibitory control was required for those trials across the entire age range included in the study.

The current results suggest that age differences in both Animal Size Stroop and magnitude comparison performance depend, at least in part, on age-related improvements in inhibitory control, but, alternatively, they could also be due to improvements in other factors, such as processing speed or number knowledge. The interaction between congruency and age on the Animal Size Stroop indicated that older children were less susceptible to size interference than younger children were and, furthermore, that the task may be too easy for older children. Fewer animals were included in this version of the task than in Szucs et al. (2009), and there were more practice trials in order to ensure 3-year-olds would be able to complete the task. However, Szucs et al. (2009) reported congruency effects in older children and adults; therefore the difficulty of the task can be adapted for use with older children. Similarly, Gilmore et al. (2015) also found significant congruency effects on reaction time on an Animal Size Stroop task in older children and adults. In summary, Experiment 2 provided support for the hypothesis that, even in very young children, performance on a measure tapping interference control (Animal Size Stroop) relates to performance on a measure traditionally thought to index number sense (non-symbolic number comparison), but, at least in young children, is increasingly better construed as a task requiring a high degree of inhibitory control.

## General discussion

Evidence was provided for a relationship between inhibitory control, as measured by the Animal Size Stroop task, and both non-symbolic magnitude comparison (Experiment 2) and a standardized measure of math achievement (Experiment 1). The role of non-symbolic magnitude comparison skills in the foundations of mathematical achievement has become highly debated (De Smedt et al., 2013) and these results provide additional support for the argument that measures of ANS precision are significantly influenced by visual parameters of non-symbolic stimuli (Gilmore et al., 2013; Szucs et al., 2013), especially for children with limited cardinality knowledge (Negen and Sarnecka, 2014). Perhaps tellingly, the youngest children in Experiment 1 failed to perform above chance (i.e., to choose systematically the most numerous displays) when a commonly used format of the magnitude comparison task was used. In Experiment 2, magnitude comparison performance was facilitated, and a robust relationship across our interference control task and magnitude comparison performance was replicated, in a sample extending to children as young as 3-years of age.

### The role of inhibitory control in magnitude comparison skills

In cognitive terms, our findings are consistent with the idea that inhibitory processes are most strongly related to mathematical operations when they require inhibiting stimulus dimensions that are in conflict with number. These results support the argument that non-symbolic comparisons require inhibitory control when perceptual features are manipulated (Gilmore et al., 2013). Similarly to what was found in previous studies (Fuhs and McNeil, 2013; Gilmore et al., 2013), performance on magnitude comparison trials when size was in conflict with number was correlated with a measure of inhibitory control, but performance on trials when size and number were correlated was not. These data, collectively, therefore point to a more specific and process-oriented relationship, rather than a more general association, between inhibitory mechanisms and numerical operations.

Ultimately, the roles of inhibitory control skills and of non-numerical dimensions as contributors to ANS precisions across ages, from childhood into adulthood, remain a highly contentious and unresolved debate. In particular, the mixed evidence (e.g., Gilmore et al., 2013; Keller and Libertus, 2015) for relationships between inhibitory control, ANS acuity, and mathematics achievement highlights how results vary based on magnitude comparison task parameters and inhibitory control measures used. We would urge these researchers to consider a possible key variable to consider in this context: the developmental timing of these interactions. An emphasis on development leads to a testable hypothesis: that inhibitory control mechanisms and non-numerical but continuous dimensions of quantities may be differentially more important when children learn about number. It is to these broader developmental theories that we now turn.

### Implications for *Developmental* models of early mathematical skills

Here we decided to assess inhibitory control as a predictor of *early* magnitude comparison skills. Our findings support the hypothesis that children become better at discerning discrete quantity with age and experience even when these conflict with other stimulus dimensions (Mix et al., 2002). In addition, these findings provide further support for their proposal that ANS theories should consider the influence of continuous quantity and inhibitory control on magnitude processing in relation to learning (e.g., Leibovich and Henik, 2013). Inhibitory control may be important for learning to attend to discrete rather than continuous properties of number. Additionally, these results suggest that children become less susceptible to conflicting size information with age and formal education, which is consistent with the finding that children were more susceptible to conflicting visual parameters on a non-symbolic magnitude comparison task than adults were (Szucs et al., 2013). In sum, the role of non-symbolic magnitude comparison skills in measuring the foundations of mathematical achievement has become highly debated (e.g., De Smedt et al., 2013; Fazio et al., 2014) and these results provide additional support for the argument that these tasks measure inhibitory control either in addition to or instead of ANS precision (Gilmore et al., 2013, but see Keller and Libertus, 2015, for contradictory results).

However, rather than delving further in this debate, our main aim here was to understand better the relationship a specific aspect of between inhibitory control (selecting task-relevant dimensions and inhibiting irrelevant dimensions) with gross mathematical achievement (Experiment 1) as well as magnitude comparison skills when faced with higher conflict from task irrelevant dimensions (Experiment 2). Beyond the role that inhibitory control might have as a concurrent constraint on mathematical operations such as magnitude comparison, however, it could be that, as Clark et al. (2013) suggested, executive skills play a more specific role in learning number skills. Perhaps the ability to inhibit irrelevant information about size and other perceptual information in non-symbolic arrays is important for helping children to learn the meaning of numbers and that, for example, “three” applies to all sets of three regardless of perceptual characteristics, something that very young children struggle with (Huang et al., 2010; Slusser and Sarnecka, 2011).

### Limitations and future directions

The current magnitude comparison task manipulated non-numerical dimensions of the stimuli in a particular way: we pitted against each other trials in which dot size and numerosity were correlated with those in which they were conflicting. We did not manipulate other non-numerical dimensions. Furthermore, incongruent and congruent trials were blocked, rather than randomly intermixed, another potential confound. However, this particular manipulation was chosen in order for our incongruent condition to be directly comparable to magnitude comparison tasks used in previous developmental studies (e.g., Rousselle et al., 2004; Halberda et al., 2008). Moreover, in Experiment 1 we found that young children (3- to 5-year-olds) did not perform above chance, which is convergent with Rousselle et al. (2004) results. This motivated us to explore the possibility that children's failure on this task may be due to immature inhibitory control but, as very young children's performance was already so heavily affected in Experiment 1, we opted against a condition in which total surface area was negatively correlated with number. An important limitation of this, and all, non-symbolic magnitude comparison tasks is the following: recent evidence suggests that performance is strongly influenced by the parameters of the stimuli and that results of comparisons with different non-symbolic stimuli do not correlate with each other (Clayton et al., 2015; Smets et al., 2015). In particular, Clayton et al. (2015) showed that adults showed a reversed congruence effect on comparisons of stimuli generated using Panamath software (Halberda et al., 2008) compared to using the method of Gebuis and Reynvoet (2012). Specifically, the Panamath stimuli do not control for convex hull across stimuli, and participants' accuracy was higher for total surface area incongruent than congruent trials, whereas with Gebuis and Reynvoet's stimuli, they found the reverse, but only for trials on which convex hull was incongruent, as they found convex hull to exert a stronger influence on performance than total surface area did (Clayton et al., 2015). As Keller and Libertus (2015) used the Panamath software and also showed this reverse congruence effect, this could account for discrepancies between their findings and the results of the current study. More broadly, this highlights challenges in drawing conclusions about exactly what non-symbolic comparison tasks are tapping into and the extent to which inhibitory control is required across studies employing differing methods of generating stimuli.

Gaining greater insights into the relationship between inhibitory control and math achievement could lead to applications for math curriculum design in early childhood. In particular, if selecting number as the relevant stimulus dimension (in particular when in conflict with other stimulus dimensions) plays a role in learning about number, then this should be emphasized in instructional regimes. The results presented here highlight the importance of inhibitory control for extracting information about number from non-symbolic arrays when faced with distracting size cues and suggest that this should be taken into consideration when assessing number skills in young children, but perhaps also when teaching them. It is possible that inhibitory control completely accounts for the relationship between non-symbolic comparison and magnitude comparison, specifically in younger children (Fazio et al., 2014). Of note, symbolic number skills have been found to be stronger predictors of maths achievement than non-symbolic skills in children over the age of five (e.g., Göbel et al., 2014). As young children have limited symbolic knowledge, evaluating relationships between symbolic knowledge and maths achievement in this age group is less straightforward. However, recent evidence suggests that cardinality knowledge mediates the relationships between ANS acuity and maths achievement, even in preschoolers (Chu et al., 2015). It remains unclear exactly how non-symbolic numerical representations are important for the acquisition of symbolic knowledge. Clarifying the mechanisms underlying these relationships and the hypothesized role of inhibitory control could be useful for informing methods of teaching about symbolic number prior to the start of formal education.

In conclusion, the two experiments presented here provide further evidence that non-symbolic magnitude comparison tasks require inhibition of irrelevant stimuli dimensions associated with continuous quantity (Gilmore et al., 2013; Szucs et al., 2013; Clayton and Gilmore, 2015), especially in young children. This finding isolates an inhibitory process related to this math skill and therefore highlights the need to test relationships between specific inhibitory processes and particular mathematical operations. In broader terms, these results also emphasize that researchers should consider the overlapping nature of executive demands with other developing skills when designing inhibitory control assessments for preschoolers. In addition to relating to magnitude comparison, performance on the Animal Size Stroop was also correlated with a more general measure of math. The specific mechanisms underlying the relationship between executive control and general math achievement remain to be explored further. In order to investigate the hypothesis that inhibitory control and other executive processes are important for *learning* about math, future work should investigate how children learn, in addition to assessing what they know, and the role played by executive functions in the process of learning itself.

### Conflict of interest statement

The authors declare that the research was conducted in the absence of any commercial or financial relationships that could be construed as a potential conflict of interest.

## References

[B1] Best J. R. Miller P. H. Naglieri J. A. (2011). Relations between executive function and academic achievement from ages 5 to 17 in a large, representative national sample. Learn. Indiv. Differ. 21, 327–336. 10.1016/j.lindif.2011.01.007 21845021 PMC3155246

[B2] Borst G. Poirel N. Pineau A. Cassotti M. Houdé O. (2012). Inhibitory control in number-conservation and class-inclusion tasks: a neo-Piagetian inter-task priming study. Cogn. Dev. 27, 283–298. 10.1016/j.cogdev.2012.02.004

[B3] Bryce D. Szũcs D. Soltész F. Whitebread D. (2011). The development of inhibitory control: an averaged and single-trial Lateralized Readiness Potential study. Neuroimage 57, 671–685. 10.1016/j.neuroimage.2010.12.006 21146618

[B4] Bull R. Espy K. A. Wiebe S. A. (2008). Short-term memory, working memory, and executive functioning in preschoolers: longitudinal predictors of mathematical achievement at age 7 years. Dev. Neuropsychol. 33, 205–228. 10.1080/87565640801982312 18473197 PMC2729141

[B5] Bull R. Espy K. A. Wiebe S. A. Sheffield T. D. Nelson J. M. (2011). Using confirmatory factor analysis to understand executive control in preschool children: sources of variation in emergent mathematic achievement. Dev. Sci. 14, 679–692. 10.1111/j.1467-7687.2010.01012.x 21676089 PMC3117199

[B6] Bull R. Lee K. (2014). Executive functioning and mathematics achievement. Child Dev. Perspect. 8, 36–41. 10.1111/cdep.12059

[B7] Bull R. Scerif G. (2001). Executive functioning as a predictor of children's mathematics ability: inhibition, switching, and working memory. Dev. Neuropsychol. 19, 273–293. 10.1207/S15326942DN1903_3 11758669

[B8] Cantrell L. Smith L. B. (2013). Open questions and a proposal: a critical review of the evidence on infant numerical abilities. Cognition 128, 331–352. 10.1016/j.cognition.2013.04.008 23748213 PMC3708991

[B9] Chu F. W. vanMarle K. Geary D. C. (2015). Early numerical foundations of young children's mathematical development. J. Exp. Child Psychol. 132, 205–212. 10.1016/j.jecp.2015.01.006 25705049

[B10] Clark C. A. C. Pritchard V. E. Woodward L. J. (2010). Preschool executive functioning abilities predict early mathematics achievement. Dev. Psychol. 46, 1176–1191. 10.1037/a0019672 20822231

[B11] Clark C. A. C. Sheffield T. D. Wiebe S. A. Espy K. A. (2013). Longitudinal associations between executive control and developing mathematical competence in preschool boys and girls. Child Dev. 84, 662–677. 10.1111/j.1467-8624.2012.01854.x 23006040 PMC3530644

[B12] Clayton S. Gilmore C. (2015). Inhibition in dot comparison tasks. ZDM 47, 759–770. 10.1007/s11858-014-0655-2

[B13] Clayton S. Gilmore C. Inglis M. (2015). Dot comparison stimuli are not all alike: the effect of different visual controls on ANS measurement. Acta Psychol. 161, 177 10.1016/j.actpsy.2015.09.007 26408864

[B14] Cragg L. Gilmore C. (2014). Skills underlying mathematics: the role of executive function in the development of mathematics proficiency. Trends in Neuroscience and Education. 3, 63–68. 10.1016/j.tine.2013.12.001

[B15] Dehaene S. (2011). The Number Sense: How the Mind Creates Mathematics, Revised and Updated Edition. New York: Oxford University Press.

[B16] De Smedt B. Noël M.-P. Gilmore C. Ansari D. (2013). How do symbolic and non-symbolic numerical magnitude processing skills relate to individual differences in children's mathematical skills? A review of evidence from brain and behavior. Trends Neurosci. Educ. 2, 48–55. 10.1016/j.tine.2013.06.001

[B17] Dunn L. M. Dunn D. M. (2009). The British Picture Vocabulary Scale. London: GL Assessment Limited.

[B18] Elliot C. D. Smith P. McCulloch K. (1996). British Ability Scales II. Windsor: NFER-Nelson.

[B19] Fazio L. K. Bailey D. H. Thompson C. A. Siegler R. S. (2014). Relations of different types of numerical magnitude representations to each other and to mathematics achievement. J. Exp. Child Psychol. 123, 53–72. 10.1016/j.jecp.2014.01.013 24699178

[B20] Friedman N. P. Miyake A. (2004). The relations among inhibition and interference control functions: a latent-variable analysis. J. Exp. Psychol. Gen. 133, 101–135. 10.1037/0096-3445.133.1.101 14979754

[B21] Fuhs M. W. McNeil N. M. (2013). ANS acuity and mathematics ability in preschoolers from low-income homes: contributions of inhibitory control. Dev. Sci. 16, 136–148. 10.1111/desc.12013 23278935

[B22] Gebuis T. Reynvoet B. (2012). The role of visual information in numerosity estimation. PLoS ONE 7:e37426. 10.1371/journal.pone.0037426 22616007 PMC3355123

[B23] Gerstadt C. Hong Y. Diamond A. (1994). The relationship between cognition and action: Performance of children 3^1/2^ –7 years old on a Stroop-like day-night test. Cognition 53, 129–153. 10.1016/0010-0277(94)90068-X 7805351

[B24] Gilmore C. Attridge N. Clayton S. Cragg L. Johnson S. Marlow N.. (2013). Individual differences in inhibitory control, not non-verbal number acuity, correlate with mathematics achievement. PLoS ONE 8:e67374. 10.1371/journal.pone.0067374 23785521 PMC3681957

[B25] Gilmore C. Attridge N. De Smedt B. Inglis M. (2014). Measuring the approximate number system in children: exploring the relationships among different tasks. Learn. Indiv. Differ. 29, 50–58. 10.1016/j.lindif.2013.10.004

[B26] Gilmore C. Keeble S. Richardson S. Cragg L. (2015). The role of cognitive inhibition in different components of arithmetic. ZDM 47, 771–782. 10.1007/s11858-014-0659-y

[B27] Ginsburg H. P. Baroody A. J. (2003). The Test of Early Mathematics Ability, 3rd Edn. Austin, TX: Pro Ed.

[B28] Göbel S. M. Watson S. E. Lervåg A. Hulme C. (2014). Children's arithmetic development: it is number knowledge, not the approximate number sense, that counts. Psychol. Sci. 25, 789–798. 10.1177/0956797613516471 24482406

[B29] Halberda J. Mazzocco M. M. Feigenson L. (2008). Individual differences in non-verbal number acuity correlate with maths achievement. Nature 455, 665–668. 10.1038/nature07246 18776888

[B30] Houdé O. (2000). Inhibition and cognitive development: object, number, categorization, and reasoning. Cogn. Dev. 15, 63–73. 10.1016/S0885-2014(00)00015-0

[B31] Huang Y. T. Spelke E. Snedeker J. (2010). When is four far more than three? Children's generalization of newly acquired number words. Psychol. Sci. 21, 600–606. 10.1177/0956797610363552 20424108 PMC3110717

[B32] Hughes C. Ensor R. Wilson A. Graham A. (2010). Tracking executive function across the transition to school: a latent variable approach. Dev. Neuropsychol. 35, 20–36. 10.1080/87565640903325691 20390590

[B33] Hurewitz F. Gelman R. Schnitzer B. (2006). Sometimes area counts more than number. Proc. Natl. Acad. Sci. U.S.A. 103, 19599–19604. 10.1073/pnas.0609485103 17159143 PMC1748271

[B34] Keller L. Libertus M. (2015). Inhibitory control may not explain the link between approximation and math abilities in kindergarteners from middle class families. Front. Psychol. 6:685. 10.3389/fpsyg.2015.00685 26052306 PMC4440905

[B35] Leibovich T. Henik A. (2013). Magnitude processing in non-symbolic stimuli. Front. Psychol. 4:375. 10.3389/fpsyg.2013.00375 23805121 PMC3691512

[B36] Lyons I. M. Nuerk H. C. Ansari D. (2015). Rethinking the implications of numerical ratio effects for understanding the development of representational precision and numerical processing across formats. J. Exp. Psychol. Gen. 144, 1021. 10.1037/xge0000094 26168037

[B37] Mazzocco M. M. Feigenson L. Halberda J. (2011). Preschoolers' precision of the approximate number system predicts later school mathematics performance. PLoS ONE 6:e23749. 10.1371/journal.pone.0023749 21935362 PMC3173357

[B38] Mix K. S. Huttenlocher J. Levine S. C. (2002). Multiple cues for quantification in infancy: is number one of them? Psychol. Bull. 128, 278–294. 10.1037//0033-2909.128.2.278 11931520

[B39] Miyake A. Friedman N. P. Emerson M. J. Witzki A. H. Howerter A. Wager T. D. (2000). The unity and diversity of executive functions and their contributions to complex “Frontal Lobe” tasks: a latent variable analysis. Cogn. Psychol. 41, 49–100. 10.1006/cogp.1999.0734 10945922

[B40] Naglieri J. A. Das J. P. (1997). Cognitive Assessment System. Itasca, IL: Riverside Publishing.

[B41] Negen J. Sarnecka B. W. (2014). Is there really a link between exact-number knowledge and approximate number system acuity in young children? Br. J. Dev. Psychol. 33, 92–105. 10.1111/bjdp.12071 25403910

[B42] Nigg J. T. (2000). On inhibition/disinhibition in developmental psychopathology: views from cognitive and personality psychology and a working inhibition taxonomy. Psychol. Bull. 126, 220–246. 10.1037//0033-2909.126.2.220 10748641

[B43] Nys J. Content A. (2012). Judgement of discrete and continuous quantity in adults: Number counts! Q. J. Exp. Psychol. A. 65, 675–690. 10.1080/17470218.2011.619661 22054280

[B44] Office for National Statistics (2010). Retrieved from: http://www.neighbourhood.statistics.gov.uk/dissemination/LeadHome.do?m=0&s=1448652758531&enc=1&nsjs=true&nsck=false&nssvg=false&nswid=1767

[B45] Poirel N. Borst G. Simon G. Rossi S. Cassotti M. Pineau A.. (2012). Number conservation is related to children's prefrontal inhibitory control: an fMRI study of a piagetian task. PLoS ONE 7:e40802. 10.1371/journal.pone.0040802 22815825 PMC3397932

[B46] Price G. R. Palmer D. Battista C. Ansari D. (2012). Nonsymbolic numerical magnitude comparison: reliability and validity of different task variants and outcome measures, and their relationship to arithmetic achievement in adults. Acta Psychol. (Amst) 140, 50–57. 10.1016/j.actpsy.2012.02.008 22445770

[B47] Rossion B. Pourtois G. (2004). Revisiting Snodgrass and Vanderwart's object pictorial set: the role of surface detail in basic-level object recognition. Perception 33, 217–236. 10.1068/p5117 15109163

[B48] Rousselle L. Noël M.-P. (2008). The development of automatic numerosity processing in preschoolers: evidence for numerosity-perceptual interference. Dev. Psychol. 44, 544–560. 10.1037/0012-1649.44.2.544 18331143

[B49] Rousselle L. Palmers E. Noël M.-P. (2004). Magnitude comparison in preschoolers: what counts? Influence of perceptual variables. J. Exp. Child Psychol. 87, 57–84. 10.1016/j.jecp.2003.10.005 14698689

[B50] Slusser E. B. Sarnecka B. W. (2011). Find the picture of eight turtles: A link between children's counting and their knowledge of number word semantics. J. Exp. Child Psychol. 110, 38–51. 10.1016/j.jecp.2011.03.006 21524422 PMC3105118

[B51] St Clair-Thompson H. L. Gathercole S. E. (2006). Executive functions and achievements in school: shifting, updating, inhibition, and working memory. Q. J. Exp. Psychol. 59, 745–759. 10.1080/17470210500162854 16707360

[B52] Smets K. Sasanguie D. Szücs D. Reynvoet B. (2015). The effect of different methods to construct non-symbolic stimuli in numerosity estimation and comparison. J. Cogn. Psychol. 27, 310–325. 10.1080/20445911.2014.996568

[B53] Steele A. Karmiloff-Smith A. Cornish K. Scerif G. (2012). The multiple subfunctions of attention: differential developmental gateways to literacy and numeracy. Child Dev. 83, 2028–2041. 10.1111/j.1467-8624.2012.01809.x 22804751

[B54] Szucs D. Nobes A. Devine A. Gabriel F. C. Gebuis T. (2013). Visual stimulus parameters seriously compromise the measurement of approximate number system acuity and comparative effects between adults and children. Front. Psychol. 4:444. 10.3389/fpsyg.2013.00444 23882245 PMC3715731

[B55] Szucs D. Soltész F. Bryce D. Whitebread D. (2009). Real-time tracking of motor response activation and response competition in a Stroop task in young children: a lateralized readiness potential study. J. Cogn. Neurosci. 21, 2195–2206. 10.1162/jocn.2009.21220 19296726

[B56] Van der Ven S. H. G. Kroesbergen E. H. Boom J. Leseman P. P. M. (2012). The development of executive functions and early mathematics: a dynamic relationship. Br. J. Educ. Psychol. 82(Pt 1), 100–119. 10.1111/j.2044-8279.2011.02035.x 22429060

[B57] Wagner J. B. Johnson S. C. (2011). An association between understanding cardinality and analog magnitude representations in preschoolers. Cognition 119, 10–22. 10.1016/j.cognition.2010.11.014 21288508

[B58] Wiebe S. A. Espy K. A. Charak D. (2008). Using confirmatory factor analysis to understand executive control in preschool children: I. Latent structure. Dev. Psychol. 44, 575–587. 10.1037/0012-1649.44.2.575 18331145

[B59] Wiebe S. A. Sheffield T. Nelson J. M. Clark C. A. C. Chevalier N. Espy K. A. (2011). The structure of executive function in 3-year-olds. J. Exp. Child Psychol. 108, 436–452. 10.1016/j.jecp.2010.08.008 20884004 PMC3033982

[B60] Wright I. Waterman M. Prescott H. Murdoch-Eaton D. (2003). A new Stroop-like measure of inhibitory function development: typical developmental trends. J. Child Psychol. Psychiatry 44, 561–575. 10.1111/1469-7610.00145 12751848

[B61] Yeniad N. Malda M. Mesman J. van IJzendoorn M. H. Pieper S. (2013). Shifting ability predicts math and reading performance in children: a meta-analytical study. Learn. Indiv. Differ. 23, 1–9. 10.1016/j.lindif.2012.10.004

